# Mammographic compression in Asian women

**DOI:** 10.1371/journal.pone.0175781

**Published:** 2017-04-18

**Authors:** Susie Lau, Yang Faridah Abdul Aziz, Kwan Hoong Ng

**Affiliations:** 1 Department of Biomedical Imaging, Faculty of Medicine, University of Malaya, Kuala Lumpur, Malaysia; 2 University of Malaya Research Imaging Centre, Faculty of Medicine, University of Malaya, Kuala Lumpur, Malaysia; Fu Jen Catholic University, TAIWAN

## Abstract

**Objectives:**

To investigate: (1) the variability of mammographic compression parameters amongst Asian women; and (2) the effects of reducing compression force on image quality and mean glandular dose (MGD) in Asian women based on phantom study.

**Methods:**

We retrospectively collected 15818 raw digital mammograms from 3772 Asian women aged 35–80 years who underwent screening or diagnostic mammography between Jan 2012 and Dec 2014 at our center. The mammograms were processed using a volumetric breast density (VBD) measurement software (Volpara) to assess compression force, compression pressure, compressed breast thickness (CBT), breast volume, VBD and MGD against breast contact area. The effects of reducing compression force on image quality and MGD were also evaluated based on measurement obtained from 105 Asian women, as well as using the RMI156 Mammographic Accreditation Phantom and polymethyl methacrylate (PMMA) slabs.

**Results:**

Compression force, compression pressure, CBT, breast volume, VBD and MGD correlated significantly with breast contact area (p<0.0001). Compression parameters including compression force, compression pressure, CBT and breast contact area were widely variable between [relative standard deviation (RSD)≥21.0%] and within (p<0.0001) Asian women. The median compression force should be about 8.1 daN compared to the current 12.0 daN. Decreasing compression force from 12.0 daN to 9.0 daN increased CBT by 3.3±1.4 mm, MGD by 6.2–11.0%, and caused no significant effects on image quality (p>0.05).

**Conclusions:**

Force-standardized protocol led to widely variable compression parameters in Asian women. Based on phantom study, it is feasible to reduce compression force up to 32.5% with minimal effects on image quality and MGD.

## Introduction

Breast compression is applied in mammography as it has several advantages including: (1) reduction of overlapping of breast tissues [1–4]; (2) reduction of scattered radiation and thereby, improves image contrast and reduces radiation dose to the breast [1–3, 5]; and (3) reduction of geometric and motion blurring [1–3]. Due to the breast size and elasticity, breast compression will result in the formation of a breast contact area between the breast and compression paddle [1]. The disadvantage of breast compression is the pain and discomfort caused in many women [1, 3, 6–15], particularly those who had undergone conservative treatment for breast cancer [14, 15].

During mammography, the compression force applied is usually controlled by the radiographers. They compress the breast based on the compression protocols [16–19], and also according to their experience and judgment on the breast tautness, breast size, and the pain tolerance of the women. The mammographic compression protocols currently available are all force-standardized, very subjective, and do not take breast size and elasticity into account [1, 3, 14, 20]. Studies revealed that the lack of consistent and objective guidelines in mammographic compression has led to large variations in both the force and pressure applied by radiographers during mammography [1, 3, 21–23]. Although there was a trend that greater compression forces were applied to women with greater breast volumes, large variation still existed even between women with similar breast volumes [3, 22]. Studies also showed that force-standardized compression protocols resulted in women with smaller breasts being subjected to higher compression pressures and hence, experienced more pain during mammography compared to women with larger breasts [3, 9, 20, 24]. Over-compression of the breast as a result of force-standardized protocols had also been reported in several studies, in which compressed breast thickness (CBT) was not reduced even when additional compression force was applied and thus, causing unnecessary increase in pain and discomfort without any benefits on image quality and radiation dose [11, 20, 23, 24].

The variation in compression force as a result of the varying levels of radiographers’ experience and compression techniques employed should be minimized as it will not only affect the standard of mammographic practice but also the consistency of the imaging procedure, particularly the radiation dose to women, image quality and cancer detection [1, 3, 21, 22]. Furthermore, the variation in the level of pain and discomfort experienced by women during mammographic compression should also be minimized as it will affect their participation in screening and diagnostic mammography [1, 3, 10–12, 21, 25]. Some researchers have recently suggested using pressure (force applied divided by breast contact area) instead of force to standardize mammographic compression [1, 3, 9, 14, 20, 24]. By inherently taking both breast size and elasticity into account, pressure-based standardization can potentially provide an objective and individualized compression to each breast, and lead to a better and more consistent mammographic compression [1, 3, 9, 14, 20, 24]. They further proposed standardizing compression pressure at 10 kPa (about 75 mmHg) because this pressure corresponds to the normal diastolic pressure in the breast, and results in similar effluence of venous blood from the breast regardless of breast size and compression view [craniocaudal (CC) or mediolateral-oblique (MLO)] [1, 3, 9, 14, 20].

Current force-standardized protocols [16–18] have largely been optimized for Caucasian women and thus, Asian women (who generally have smaller breasts) are subjected to protocols that might not be suitable for them. To the best of our knowledge, most studies on mammographic compression so far were carried out on Caucasian women [1, 3, 9, 14, 20, 24], and only one study was reported on Asian women for screen-film mammography [8]. In addition, as opposed to screen-film mammography, digital mammography has the advantage of the digital images being intensively processed to better show breast lesions and cancers compared to film images [26–30], hence compression force applied in digital mammography can potentially be reduced to lower the risk of over-compression and pain in women during mammography. We want to contribute to the discussion and data on mammographic compression with this study on Asian women. Hence, in this study, we aimed to: (1) investigate the mammographic compression practice at our center by analyzing the variability of compression parameters and other relevant imaging parameters within and between Asian women; and (2) evaluate the impacts of reducing compression force might have on image quality and mean glandular dose (MGD) in Asian women based on phantom study.

## Materials and methods

This study was approved by the Medical Ethics Committee of the University of Malaya Medical Centre (Reference No. 1031.13), Malaysia. Since all the images and data used in this study were anonymized, and no personal identifying information was used, the need for informed consent was waived by the committee. This study complies with the committee requirements on the use of human subject data for research.

### Clinical study

#### Study population and digital mammograms

In this part of the study, we retrospectively collected 15818 “For Processing” raw digital mammograms (CC and MLO views) acquired on three different mammography systems (GE Essential, Siemens Novation and Hologic Selenia) from 3772 Asian women aged 35–80 years (mean: 57±9 years) who underwent screening or diagnostic mammography between Jan 2012 and Dec 2014 at our center. The radiographers at our center were trained and instructed to compress the breast until it is taut or to the degree of pain which is intolerable to the women, whichever comes first, without providing a specific target force.

#### Image and data processing

All the mammograms were processed using Volpara Version 1.5.1 (Matakina Technology Limited, New Zealand) to extract compression parameters during mammography including compression force applied and CBT from the DICOM header of the images. The breast contact area between the breast and compression paddle was computed by Volpara based on the total breast area segmented from the background in the images. The compression pressure was derived from dividing compression force by breast contact area. Other parameters including breast volume, volumetric breast density (VBD) and MGD were also computed by Volpara from the images.

### Phantom study

#### Rationale for carrying out phantom study

Some researchers found that many women experienced breast pain from 12.0 daN compression force, and reducing compression force to 9.0 daN was more acceptable and tolerable to the women in their film-screen mammography study [8]. Hence, in this part of the study, we investigated the potential impacts on image quality and MGD for digital mammography in Asian women when reducing the compression force from 12.0 daN to 9.0 daN.

#### Study subjects and compressed breast thicknesses

In order to determine how much the CBT would increase when compression force is reduced from 12.0 daN to 9.0 daN, we compared the CBTs of each breast at 12.0 daN and 9.0 daN. After explaining the nature of the procedure to the women, CBTs of 105 Asian women aged 24–78 years (mean: 52±11 years) who underwent screening and diagnostic mammography between March and May 2015 at our center were recorded. All mammography procedures for these women were still carried out as per the normal clinical routine, except the CBTs of each breast at 9.0 daN and 12.0 daN were recorded, and no exposure was performed during the recording of CBTs at these two compression forces. For the actual image acquisition, no intervention was done, and the radiographers compressed the breast as per their normal clinical routine until the breast was taut. The mammograms of these women were processed with Volpara for VBD, and classified into different density categories [according to Breast Imaging-Reporting and Data System (BI-RADS) density classification] [31].

#### Image quality and mean glandular dose assessment

For investigating the effects of the increase in CBT caused by the reduction of compression force from 12.0 daN to 9.0 daN on image quality and MGD, the RMI156 Mammographic Accreditation Phantom was used to simulate a typical 4.2 cm of compressed human breast (composed of 50% adipose tissue and 50% glandular tissue). The various test features embedded in the phantom allow for the simulation of different types of breast lesions (fibers, masses and calcifications). Additionally, slabs of 10.2 cm by 10.8 cm polymethyl methacrylate (PMMA) of different thicknesses were used to simulate different thicknesses of breast tissue.

The RMI156 phantom was first exposed using the Hologic Selenia system with the two most frequently used anode/filter combinations and tube voltages (kVp), i.e., tungsten/rhodium combination (W/Rh) at 28 kVp, and tungsten/silver combination (W/Ag) at 30 kVp. The phantom was then exposed with PMMA slabs of different thicknesses (1–6 mm, with 1 mm increment) added between the phantom and compression paddle to simulate the increase in CBT. Two radiologists (A and B) and two radiographers (C and D) were then asked to score the phantom image quality when no PMMA slab was added up until 6 mm PMMA slabs were added based on the test features (fibers, masses and calcifications) they could observe in the images displayed on a diagnostic monitor. A description of how the image quality was scored using the RMI156 Mammographic Accreditation Phantom is available in references [18] and [32]. All the observers were all professionally trained in their respective fields. Radiologists A and B have more than 17 and 7 years of professional experience in radiology, respectively, and both Radiographers C and D have more than 5 years of professional experience in radiography, respectively. MGD for each exposure was also calculated.

### Statistical analyses

For the clinical study, scatter plots and box plots were used to visualize and compare the compression parameters (compression force, compression pressure, CBT and breast contact area), and other relevant parameters (breast volume, VBD and MGD). All parameters (except breast contact area) were plotted against breast contact area on the scatter plots. Spearman's correlation coefficients (*ρ*) for the relationships between these parameters and breast contact area were computed. Wilcoxon signed rank test was used to assess the statistical difference of each parameter between the CC and MLO views within the women. A p-value of less than 0.05 was deemed statistically significant. The relative standard deviation (RSD) was used to assess the variability of each parameter amongst the women.

For the phantom study, Wilcoxon signed rank test was used to assess the significant difference in the women’s CBTs when the compression force was reduced from 12.0 daN to 9.0 daN, as well as the significant difference in the phantom image quality scores with and without PMMA slabs added during the exposures. A p-value of less than 0.05 was deemed statistically significant. In addition, linearly weighted kappa statistic was used to assess the agreement of image quality scores between the four observers (A, B, C and D) based on all their fiber, mass, calcification and total scores. Weighted kappa of 0.00–0.20 was interpreted as slight agreement, 0.21–0.40 as fair agreement, 0.41–0.60 as moderate agreement, 0.61–0.80 as substantial agreement and 0.81–1.00 as almost perfect agreement.

Statistical analyses were performed using SPSS (Version 16.0, SPSS Inc., USA) and MedCalc (Version 12.5, MedCalc Software, Belgium).

## Results

### Clinical study

**Fig 1:**
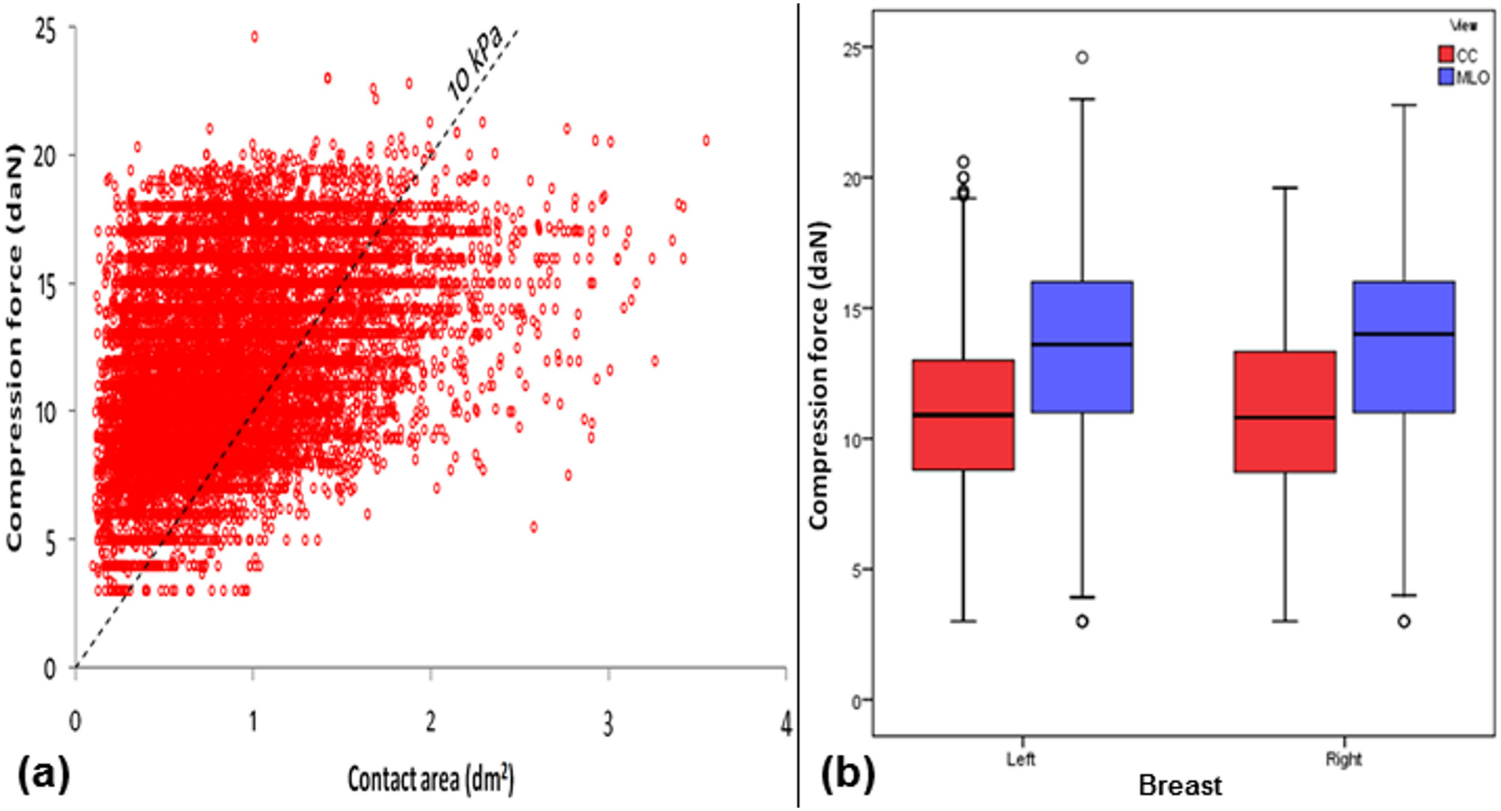
(a) Scatter plot showing compression force against contact area (N = 15818). (b) Box plot showing compression force for the left CC (n = 3897), left MLO (n = 3954), right CC (n = 3960) and right MLO views (n = 4007).

**Fig 2:**
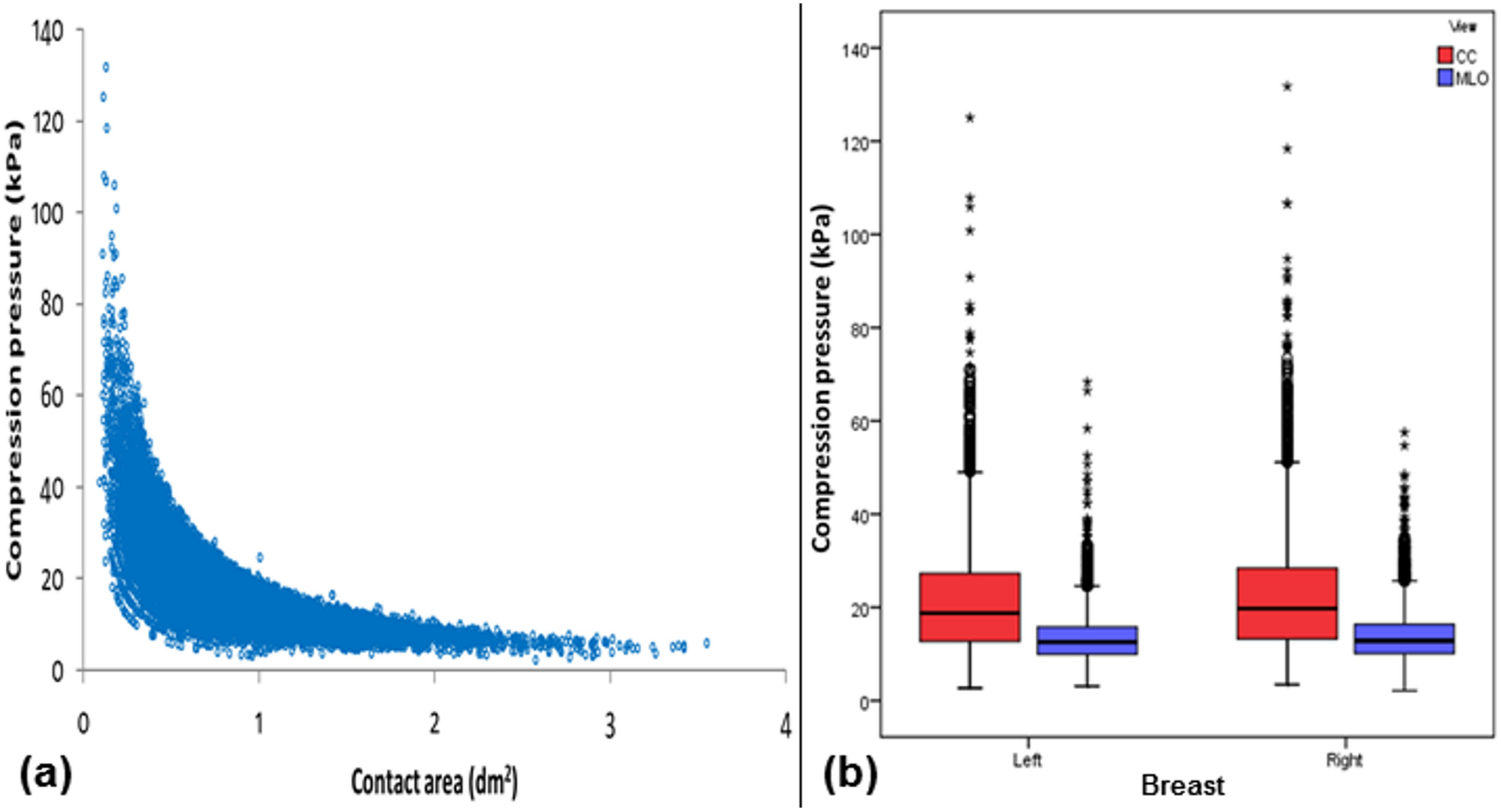
(a) Scatter plot showing compression pressure against contact area (N = 15818). (b) Box plot showing compression pressure for the left CC (n = 3897), left MLO (n = 3954), right CC (n = 3960) and right MLO views (n = 4007).

**Fig 3:**
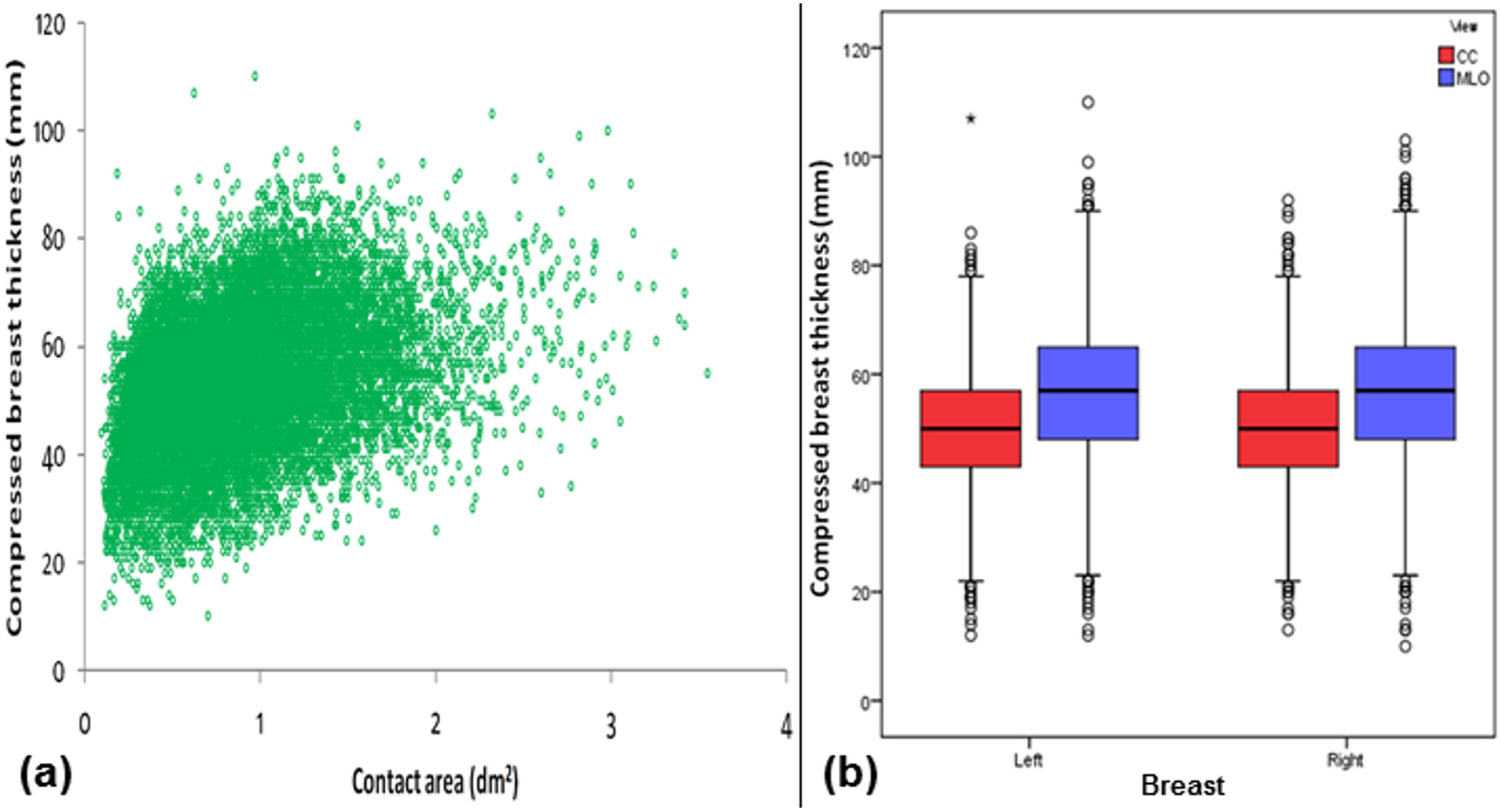
(a) Scatter plot showing compressed breast thickness against contact area (N = 15818). (b) Box plot showing compressed breast thickness for the left CC (n = 3897), left MLO (n = 3954), right CC (n = 3960) and right MLO views (n = 4007).

**Fig 4:**
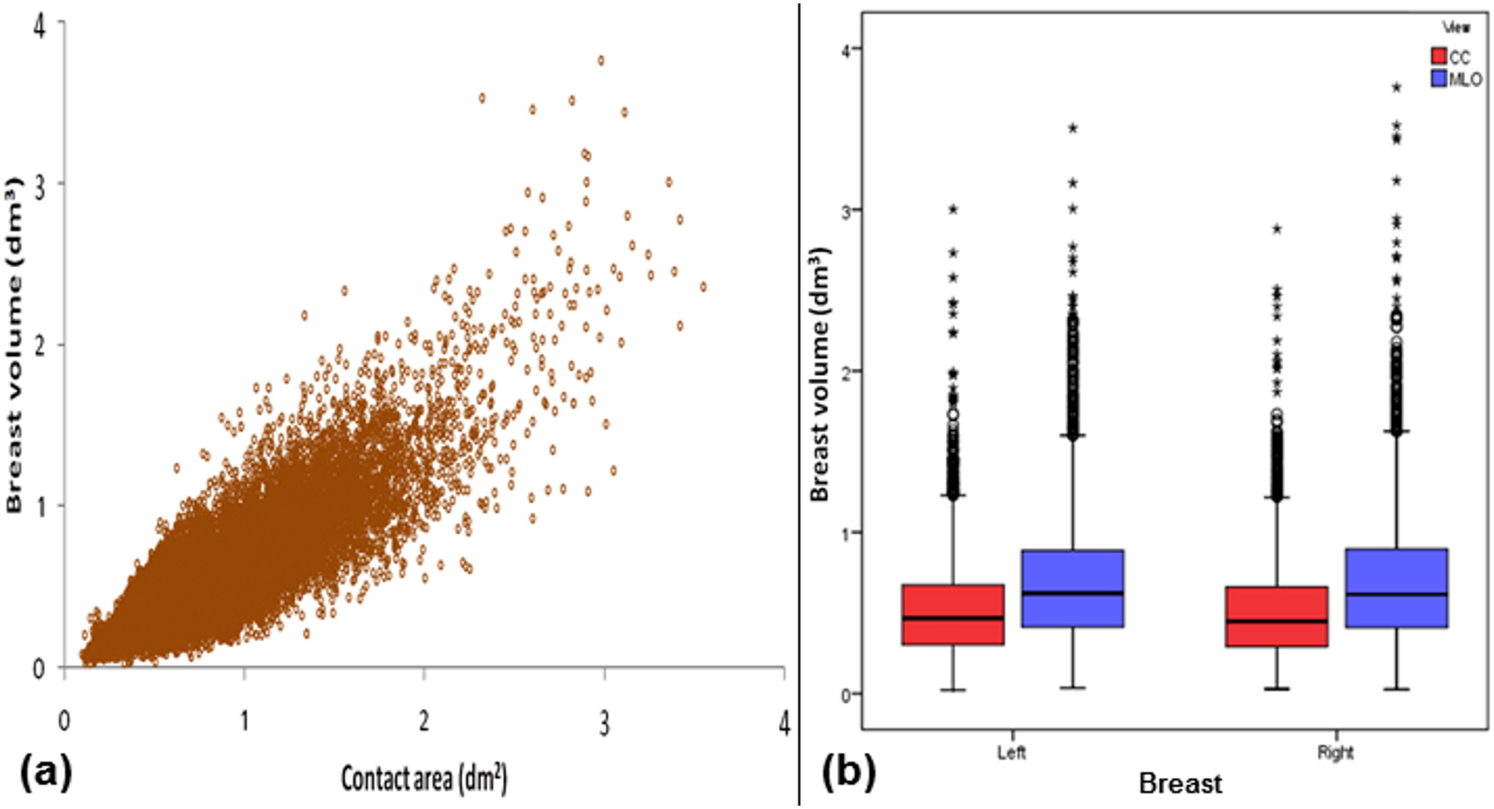
(a) Scatter plot showing breast volume against contact area (N = 15818). (b) Box plot showing breast volume for the left CC (n = 3897), left MLO (n = 3954), right CC (n = 3960) and right MLO views (n = 4007).

**Fig 5:**
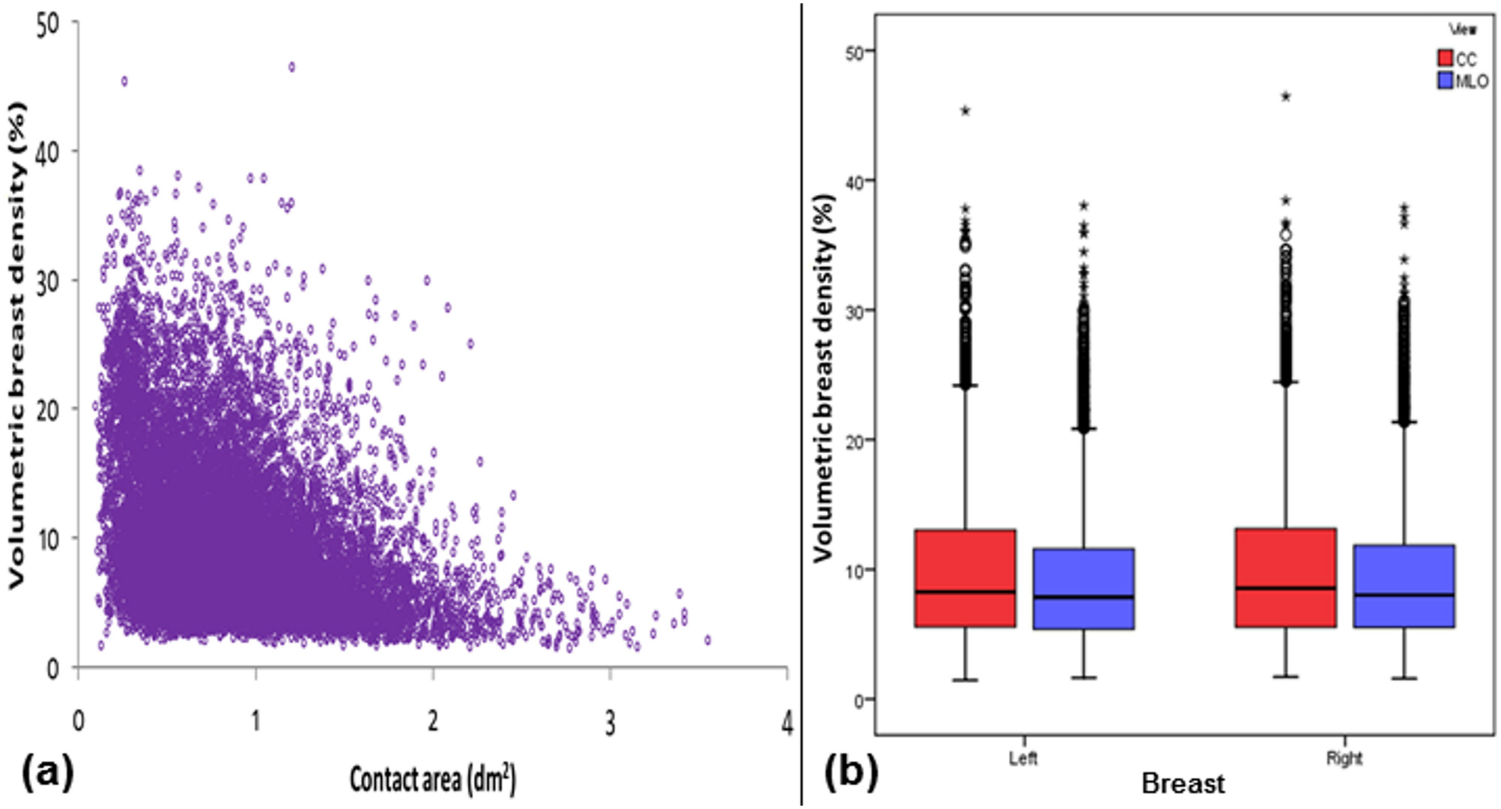
(a) Scatter plot showing volumetric breast density against contact area (N = 15818). (b) Box plot showing volumetric breast density for the left CC (n = 3897), left MLO (n = 3954), right CC (n = 3960) and right MLO views (n = 4007).

**Fig 6:**
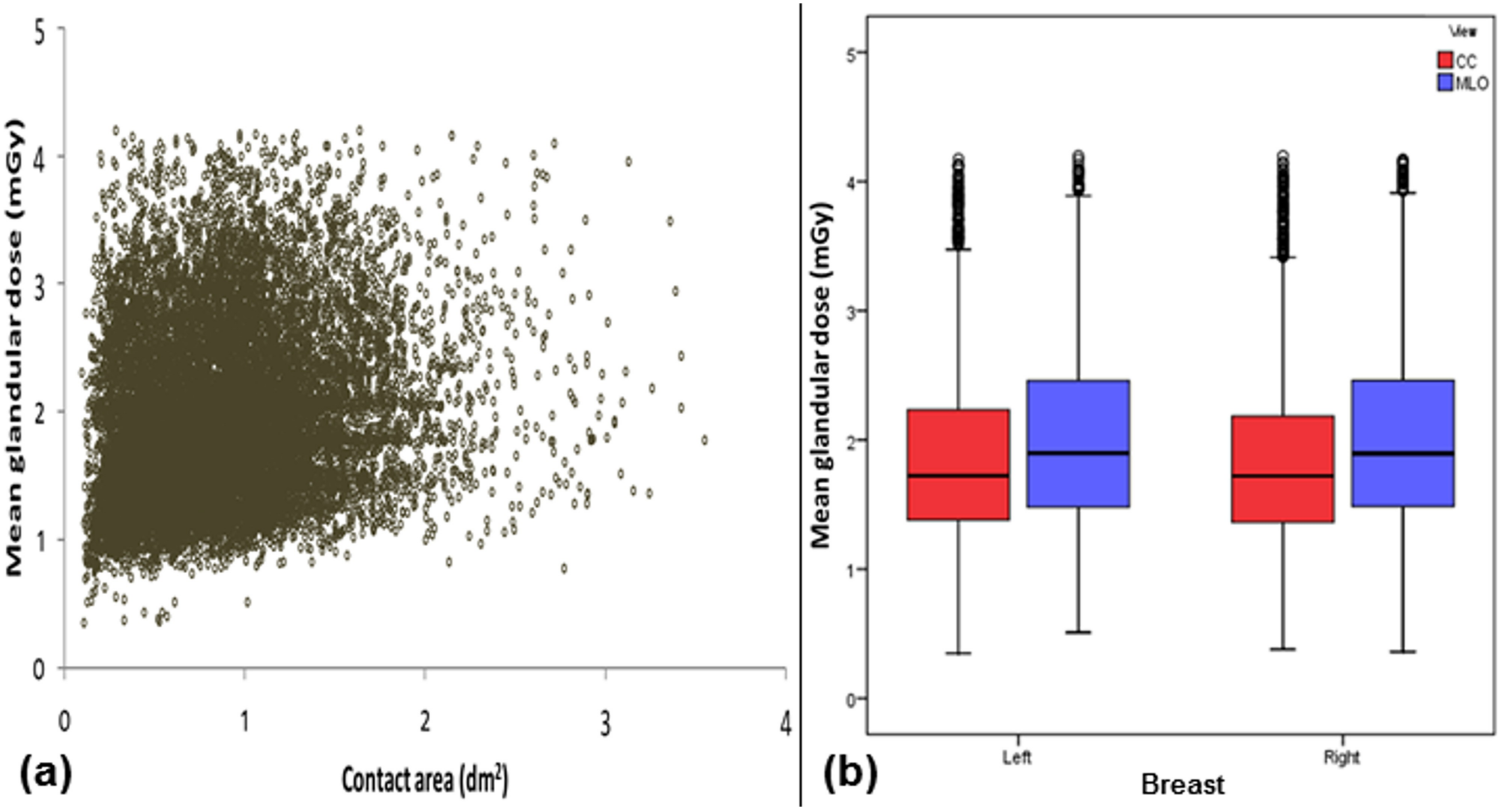
(a) Scatter plot showing mean glandular dose against contact area (N = 15818). (b) Box plot showing mean glandular dose for the left CC (n = 3897), left MLO (n = 3954), right CC (n = 3960) and right MLO views (n = 4007).

**Fig 7:**
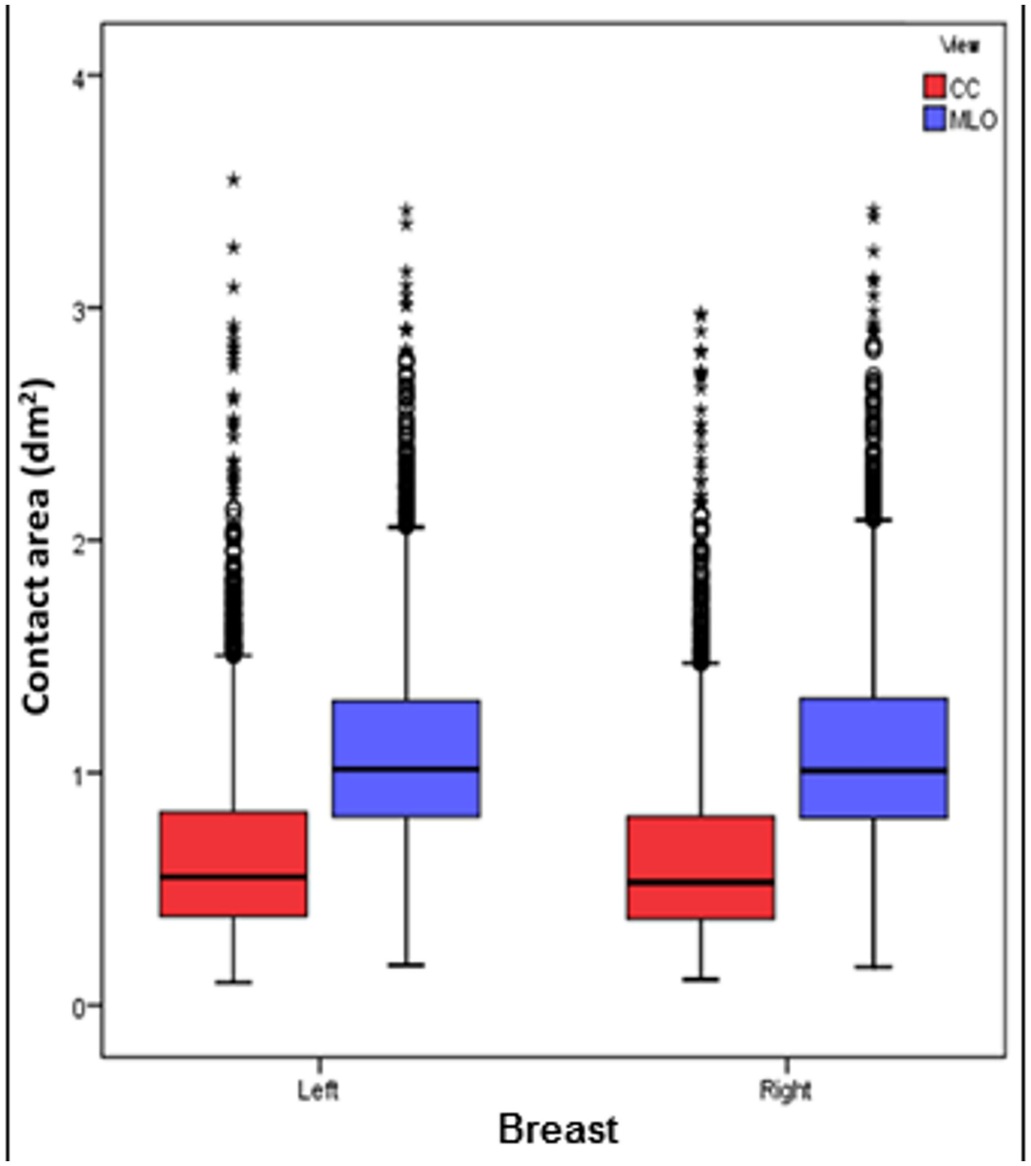
Box plot showing contact area for the left CC (n = 3897), left MLO (n = 3954), right CC (n = 3960) and right MLO views (n = 4007).

In part (a) of Figs 1–6, scatter plot is used to show the relationship between each investigated parameter (including compression force, compression pressure, CBT, breast volume, VBD and MGD) and breast contact area; whereas in part (b), box plot is used to visualize and compare each corresponding parameter in different mammographic views. Additionally, in Fig 7, box plot is used to show the comparison of the breast contact area in different mammographic views. Spearman's correlation coefficients (*ρ*) for the relationships between the parameters and breast contact area are summarized in Table 1. There were very strong significant correlations between compression pressure and breast contact area (*ρ* = -0.82, p<0.0001) [Fig 2(a)], and between breast volume and breast contact area (*ρ* = 0.82, p<0.0001) [Fig 4(a)], respectively. These were expected since both compression pressure and breast volume were derived from breast contact area $\left(\text{compression}\;\text{pressure}=\frac{\text{compression}\;\text{force}}{\text{breast}\;\text{contact}\;\text{area}}\;\;\text{and}\;\text{breast}\;\text{volume}=\text{breast}\;\text{contact}\;\text{area}\times \text{CBT}\right)$. Compression force, CBT and VBD showed moderate significant correlation with breast contact area (*ρ* = 0.44, 0.37 and -0.36, respectively, p<0.0001) [Figs 1(a), 3(a) and 5(a), respectively]. Additionally, MGD showed weak but significant correlation with breast contact area (*ρ* = 0.22, p<0.0001) [Fig 6(a)].

**Table 1:** Correlation between compression force, compression pressure, compressed breast thickness, breast volume, volumetric breast density and mean glandular dose with contact area (N = 15818).

Correlation	*ρ* (95% CI)	Significance level*
compression force—breast contact area	0.44 (0.43, 0.46)	p<0.0001
compression pressure—breast contact area	-0.82 (-0.83, -0.82)	p<0.0001
CBT—breast contact area	0.37 (0.36, 0.39)	p<0.0001
Breast volume—breast contact area	0.82 (0.81, 0.82)	p<0.0001
VBD—breast contact area	-0.36 (-0.37, -0.34)	p<0.0001
MGD—breast contact area	0.22 (0.21, 0.24)	p<0.0001

CBT = compressed breast thickness, VBD = volumetric breast density, MGD = mean glandular dose, *ρ* = Spearman’s correlation coefficient, CI = confidence interval.

*Spearman’s correlation.

The mean±SD, RSD and median of compression force, compression pressure, CBT, breast contact area, breast volume, VBD and MGD for all mammograms, CC and MLO views, as well as the p-value from Wilcoxon signed rank test to assess the statistical difference of each parameter between the CC and MLO views are summarized in Table 2. The high RSDs for compression force (CC: 29.5%, MLO: 24.1%) and compression pressure (CC: 57.0%, MLO: 39.7%) indicate that the currently practiced force-standardized protocol has resulted in large variations in compression force applied amongst the women, and the variations in the compression pressure applied were even larger. Wilcoxon signed rank test also showed that the compression force applied for MLO view (median: 14.0 daN) was significantly higher than CC view (median: 10.9 daN) within the women (p<0.0001), whereas the compression pressure applied for CC view (median: 19.2 kPa) was significantly higher than MLO view (median: 12.7 kPa) (p<0.0001). It should be noted that the maximum compression pressure used was estimated to be 131.7 kPa, which occurred when the right CC view image of one of the subjects was acquired [Fig 2(a) and 2(b)].

**Table 2:** Summary of the mean, standard deviation, relative standard deviation and median of each parameter for all mammograms (N = 15818), CC (n = 7857) and MLO (n = 7961) views, as well as the p-value from Wilcoxon test to assess the statistical difference between the CC and MLO views.

Parameter	Overall (N = 15818)			CC view (n = 7857)			MLO view (n = 7961)			p-value from Wilcoxon test
	Mean±SD	RSD (%)	Median (95% CI)	Mean±SD	RSD (%)	Median (95% CI)	Mean±SD	RSD (%)	Median (95% CI)	
Compression force (daN)	12.22±3.45	28.22	12.00 (12.00, 12.00)	11.03±3.26	29.54	10.90 (10.70, 11.00)	13.40±3.22	24.06	14.00 (13.80, 14.00)	<0.0001
Compression pressure (kPa)	17.77±10.51	59.13	14.81 (14.66, 14.94)	22.00±12.53	56.95	19.24 (18.95, 19.56)	13.59±5.40	39.72	12.73 (12.60, 12.86)	<0.0001
CBT (mm)	53.17±12.18	22.92	53.00 (53.00, 53.00)	49.84±10.47	21.00	50.00 (50.00, 50.00)	56.45±12.85	22.77	57.00 (56.00, 57.00)	<0.0001
Breast contact area (dm^2^)	0.87±0.46	52.73	0.81 (0.81, 0.82)	0.65±0.39	59.56	0.54 (0.53, 0.55)	1.10±0.42	38.04	1.01 (1.01, 1.02)	<0.0001
Breast volume (dm^3^)	0.60±0.36	59.88	0.53 (0.53, 0.54)	0.51±0.30	58.58	0.46 (0.45, 0.46)	0.69±0.39	56.76	0.62 (0.61, 0.63)	<0.0001
VBD (%)	9.76±5.82	59.58	8.14 (8.05, 8.25)	10.17±6.19	60.84	8.42 (8.25, 8.58)	9.36±5.40	57.66	7.94 (7.81, 8.05)	<0.0001
MGD (mGy)	1.93±0.66	34.32	1.79 (1.78, 1.80)	1.84±0.64	34.72	1.72 (1.71, 1.74)	2.01±0.67	33.40	1.90 (1.87, 1.91)	<0.0001

CC = craniocaudal, MLO = mediolateral-oblique, SD = standard deviation, RSD = relative standard deviation, CI = confidence interval, CBT = compressed breast thickness, VBD = volumetric breast density, MGD = mean glandular dose.

From the clinical study, we found that the overall median compression force for our study population was approximately 12.0 daN. However, based on the overall median breast contact area (0.81 dm^2^) and the proposed 10 kPa pressure-standardized protocol, the corresponding median compression force should be approximately 8.1 daN [Fig 1(a)]. Consequently, we carried out a phantom study to investigate the impacts of reducing compression force on image quality and MGD.

### Phantom study

Table 3 summarizes the CBTs at 9.0 daN and 12 daN, as well as the difference in CBT when the compression force was reduced from 12.0 daN to 9.0 daN for the 105 women (342 mammograms) according to the mammographic view, age group and BI-RADS density category. The mean±SD of CBT for these women during the actual image acquisitions was 54.6±12.2 mm. Overall, the mean increase in CBT was 3.3±1.4 mm when compression force was reduced from 12.0 daN (52.9±12.1 mm) to 9.0 daN (56.2±12.1 mm). Paired t-test showed that the CBTs were highly significantly different when the compression force was reduced from 12.0 daN to 9.0 daN for both CC and MLO views (p<0.0001). The difference in CBT was larger for the MLO view (3.5±1.9 mm) as compared to CC view (3.1±1.9 mm). Paired t-test also showed that the CBTs were highly significantly different when the compression force was reduced from 12.0 daN to 9.0 daN for the different age groups and BI-RADS density categories (p<0.0001 for all).

**Table 3:** Summary of compressed breast thicknesses of the women according to the mammographic view, age group and BI-RADS density category.

	Mean±SD for CBT (mm)										
	Overall (N = 342)	Mammographic view		Age (years)				BI-RADS density category			
		CC (n = 175)	MLO (n = 167)	<40 (n = 38)	40–49 (n = 86)	50–59 (n = 130)	≥60 (n = 88)	1 (n = 34)	2 (n = 103)	3 (n = 139)	4 (n = 66)
**CBT at 9.0 daN**	56.2±12.1	53.0±11.1	59.7±12.3	58.0±11.3	54.1±15.4	57.2±10.1	51.8±11.3	60.1±6.3	57.8±11.6	55.4±12.1	52.3±9.1
**CBT at 12.0 daN**	52.9±12.1	49.9±11.2	56.0±12.3	54.7±11.8	52.7±11.5	54.0±10.5	48.3±11.3	56.7±6.8	54.6±11.8	52.1±12.2	48.8±9.1
**Difference in CBT**	3.3±1.4	3.1±1.9	3.5±1.9	3.3±1.8	3.3±1.3	3.3±1.5	3.5±1.3	3.4±1.6	3.1±1.2	3.3±1.2	3.5±2.0

CC = craniocaudal, MLO = mediolateral-oblique, SD = standard deviation, CBT = compressed breast thickness, BI-RADS = Breast Imaging-Reporting and Data System.

To be more conservative, we compared the image quality scored by the radiologists and radiographers when the phantom was exposed without and with 5 mm PMMA slabs added. The results for W/Rh at 28 kVp and W/Ag at 30 kVp are shown in Tables 4 and 5, respectively. Tables 6 and 7 show the weighted kappa values for the agreement of image quality scores between the four observers based on all their fiber, mass, calcification and total scores when the RMI156 phantom was exposed both with and without 5 mm of PMMA slabs added for W/Rh at 28 kVp and W/Ag at 30 kVp, respectively. Weighted kappa statistic indicated that the agreement of image quality scores assessed by the four observers ranged from moderate to almost perfect agreement for W/Rh at 28 kVp, and substantial to almost perfect agreement for W/Ag at 30 kVp. Wilcoxon signed rank test revealed that there was no significant difference in the fiber, mass, calcification and total scores when the phantom was exposed without and with 5 mm of PMMA slabs added for both W/Rh at 28 kVp and W/Ag at 30 kVp (p>0.05), indicating that the image quality was similar whether the phantom was exposed without or with the 5 mm PMMA slabs added. Hence, we expect the increase of 3.3±1.4 mm in CBT in the women would have limited impact on image quality.

**Table 4:** Mean, standard deviation and range for the image quality scores of the radiologists and radiographers when the RMI156 phantom was exposed without and with 5 mm of PMMA slabs added for W/Rh at 28 kVp.

	Fiber score (Mean±SD)	Range	Mass score (Mean±SD)	Range	Calcification score (Mean±SD)	Range	Total score (Mean±SD)	Range
**Without PMMA slabs added**	5.4±0.5	1.0	4.8±0.3	0.5	4.6±0.3	0.5	14.8±1.0	2.0
**With 5 mm PMMA slabs added**	5.1±0.3	0.5	4.4±0.3	0.5	4.3±0.3	0.5	13.8±0.3	0.5
**p-value from Wilcoxon test**	p = 0.16	-	p = 0.08	-	p = 0.18	-	p = 0.11	-

PMMA = polymethyl methacrylate, SD = standard deviation.

**Table 5:** Mean, standard deviation and range for the image quality scores of the radiologists and radiographers when the RMI156 phantom was exposed without and with 5 mm of PMMA slabs added for W/Ag at 30 kVp.

	Fiber score (Mean±SD)	Range	Mass score (Mean±SD)	Range	Calcification score (Mean±SD)	Range	Total score (Mean±SD)	Range
**Without PMMA slabs added**	5.4±0.5	1.0	4.6±0.3	0.5	4.4±0.3	0.5	14.4±0.5	1.0
**With 5 mm PMMA slabs added**	5.0±0.0	0.0	4.4±0.3	0.5	4.3±0.3	0.5	13.6±0.3	0.5
**p-value from Wilcoxon test**	p = 0.18	-	p = 0.16	-	p = 0.32	-	p = 0.11	-

PMMA = polymethyl methacrylate, SD = standard deviation.

**Table 6:** Weighted kappa value for the agreement of image quality assessment between the four observers based on all their fiber, mass, calcification and total scores when the RMI156 phantom was exposed both with and without 5 mm of PMMA slabs added for W/Rh at 28 kVp.

Observer	Weighted kappa (95% CI)			
	Radiologist A	Radiologist B	Radiographer C	Radiographer D
**Radiologist A**	-	0.76 (0.62, 0.91)	0.58 (0.32, 0.83)	0.54 (0.34, 0.75)
**Radiologist B**	-	-	0.67 (0.51, 0.83)	0.67 (0.46, 0.87)
**Radiographer C**	-	-	-	0.81 (0.61, 1.00)

CI = confidence interval.

**Table 7:** Weighted kappa value for the agreement of image quality assessment between the four observers based on all their fiber, mass, calcification and total scores when the RMI156 phantom was exposed both with and without 5 mm of PMMA slabs added for W/Ag at 30 kVp.

Observer	Weighted kappa (95% CI)			
	Radiologist A	Radiologist B	Radiographer C	Radiographer D
**Radiologist A**	-	0.80 (0.65, 0.94)	0.69 (0.56, 0.82)	0.68 (0.49, 0.87)
**Radiologist B**	-	-	0.67 (0.51, 0.83)	0.67 (0.46, 0.87)
**Radiographer C**	-	-	-	0.81 (0.61, 1.00)

CI = confidence interval.

**Fig 8:**
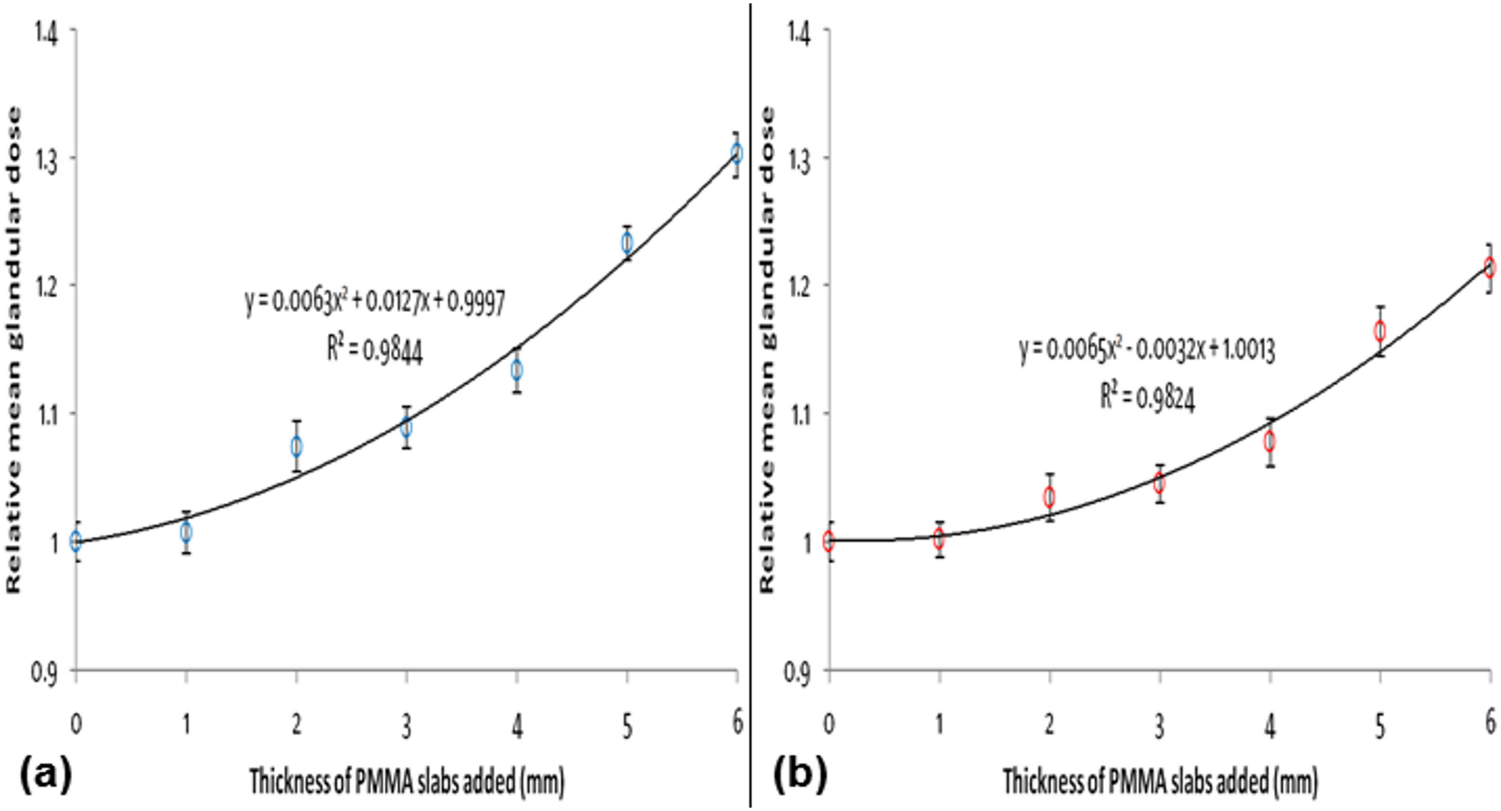
Relationship between the relative mean glandular dose and the thickness of PMMA slabs added to the RMI156 phantom exposed with (a) W/Rh at 28 kVp and (b) W/Ag at 30 kVp.

The MGD estimated for the RMI156 phantom exposed without any PMMA slab added for W/Rh at 28 kVp and W/Ag at 30 kVp were 1.3 mGy and 1.0 mGy, respectively. Fig 8(a) and 8(b) show the relationships between the relative MGDs and the thicknesses of PMMA slabs added to the phantom exposed with W/Rh at 28 kVp and W/Ag at 30 kVp, respectively. From the established quadratic equations for W/Rh at 28 kVp and W/Ag at 30 kVp [shown in Fig 8(a) and 8(b)], the estimated increases in MGD would be about 11.0% and 6.2%, respectively when the mean CBT increased by 3.3 mm (while the compression force decreased from 12.0 daN to 9.0 daN), which is minimal. The MGD per view would still be well under the dose recommended by various widely used standard protocols (less than 3 mGy per view) [16–18]. Therefore, the increase in CBT caused by the decrease in compression force from 12.0 daN to 9.0 daN has limited effects on MGD.

## Discussion

Currently available force-standardized protocols are very subjective and provide no clear guidelines on the suitable amount of compression force to be used for individual breasts and hence, resulted in widely variable mammographic compression force and compression pressure in Caucasian women [1, 3, 21–23]. In this study, we investigated: (1) the variability of compression parameters (compression force, compression pressure, CBT and breast contact area) and other relevant parameters (breast volume, VBD and MGD) between and within Asian women during mammography; and (2) the effects of reducing compression force on image quality and MGD based on phantom study in Asian women.

Very strong and highly significant correlations were observed between compression pressure and breast contact area, and also between breast volume and breast contact area because both compression pressure and breast volume were derived from breast contact area. There were moderate correlations between compression force, CBT and VBD with breast contact area, and weak correlation between MGD with breast contact area, but all correlations are highly significant. Similar to previous studies reported on Caucasian women [1, 3, 21–23], current force-standardized protocols had also led to highly significant variable compression force and compression pressure between and within Asian women (RSD≥24.1%, p<0.0001). Although the mean compression force in our study was comparable to previous studies [1, 3, 20, 21], the compression pressure is generally higher than those reported in Caucasian women [1, 20]. This is possibly because the breast contact area of the Asian women in our study is generally smaller than Caucasian women [3, 20]. The maximum compression pressure observed in our study was 131.7 kPa, which was surprisingly high. As expected, the mean CBT of the Asian women in our study was smaller than those reported for Caucasian women [1, 3, 20], whereas the mean VBD were higher than those of Caucasian women [1]. Furthermore, the MGD estimated in this study was comparable to those previously reported [1, 20, 33].

Based on the proposed 10 kPa the pressure-standardized protocol and our overall median breast contact area (0.81 dm^2^), we estimated that the median compression force (8.1 daN) should be approximately 32.5% lower than our current practice (12.0 daN). We found that although reducing the compression force from 12.0 daN to 9.0 daN could potentially reduce the pain and discomfort to the women, it resulted in an overall average increase of 3.3±1.4 mm in the CBT, which was comparable to another study carried out on Asian women [8]. Moreover, the phantom study revealed that an increase of 5 mm (and thus, 3.3±1.4 mm) in CBT have limited impact on image quality, and the increase of 3.3±1.4 mm in CBT has also limited impact on MGD.

This study has some limitations. Firstly, our results may be biased since the data was collected from only one center. A multi-center study, with data collected from different centers from different Asian regions, would be ideal for generalizing our findings. Secondly, the data set was assumed to represent the normal routine in this region as the data set was large and the mammograms were acquired by a large number of radiographers (25 of them). Thirdly, calibration errors in mammography systems may introduce errors in compression force measurement. However, when compared to the wide variations and significant differences in compression force and compression pressure amongst the women in our study, the calibration errors are minimal. Lastly, the RMI156 phantom and PMMA slabs used to simulate the compressed breast and breast tissue, respectively are both incompressible. Ideally, breast tissue-equivalent attenuation materials which are compressible should be used for better and more realistic measurements. Nevertheless, the various embedded test features in the phantom were useful for simulating different types of breast lesions (fibers, masses and calcifications), and the quantitative results obtained for CBT, image quality and MGD provide useful information.

In summary, currently available force-standardized protocols do not take breast size and elasticity into account, and led to widely variable compression parameters, particularly compression force and compression pressure, not only amongst Caucasian women but also amongst Asian women. Additionally, these force-standardized protocols have largely been optimized for Caucasian women, thus Asian women who generally have smaller breasts are subjected to protocols that might not be suitable for them. Our results indicated that it is feasible to reduce compression force in Asian women with limited impacts on image quality and MGD in digital mammography.

## Supporting information

S1 Data Data including subject age, breast side, mammographic view, compression force, compression pressure, compressed breast thickness, breast contact area, breast volume, volumetric breast density and mean glandular dose recorded for the 15818 “For Processing” raw digital mammograms acquired from 3772 Asian women in the clinical study. (XLSX)

S2 Data Data including subject age, BI-RADS density category, compression force, compressed breast thickness and mammographic view recorded for the 105 Asian women in the phantom study. (XLSX)

S3 Data Data including fibre, mass, calcification and total scores recorded for the radiologists and radiographers when the RMI156 phantom was exposed without and with 5 mm of PMMA slabs added for W/Rh at 28 kVp and W/Ag at 30 kVp. (XLSX)

## Abbreviations

ρ: Spearman's correlation coefficient
BI-RADS: Breast Imaging-Reporting and Data System
CBT: Compressed breast thickness
CC: Craniocaudal
CI: Confidence interval
kVp: Tube voltage
MGD: Mean glandular dose
MLO: Mediolateral-oblique
p: p-value
PMMA: Polymethyl methacrylate
RSD: Relative standard deviation
SD: Standard deviation
VBD: Volumetric breast density
